# Progesterone from ovulatory menstrual cycles is an important cause of breast cancer

**DOI:** 10.1186/s13058-023-01661-0

**Published:** 2023-05-30

**Authors:** Herjan J. T. Coelingh Bennink, Iman J. Schultz, Marcus Schmidt, V. Craig Jordan, Paula Briggs, Jan F. M. Egberts, Kristina Gemzell-Danielsson, Ludwig Kiesel, Kirsten Kluivers, Jan Krijgh, Tommaso Simoncini, Frank Z. Stanczyk, Robert D. Langer

**Affiliations:** 1 Pantarhei Bioscience BV, P.O. Box 464, 3700 AL Zeist, The Netherlands; 2 Department of Obstetrics and Gynaecology, University Medical Center Mainz, Mainz, Germany; 3 Department of Breast Medical Oncology, University of Texas MD Anderson Cancer Center, Houston, TX USA; 4 Sexual and Reproductive Health, Liverpool Women’s NHS Foundation Trust, Liverpool, UK; 5 Terminal 4 Communications, Hilversum, The Netherlands; 6 Department of Women’s and Children’s Health, Karolinska Institutet, Stockholm, Sweden; 7 Department of Gynaecology and Obstetrics, University of Münster, Münster, Germany; 8 Department of Obstetrics and Gynaecology, Radboud University Medical Centre, Nijmegen, The Netherlands; 9 Department of Clinical and Experimental Medicine, University of Pisa, Pisa, Italy; 10 Department of Obstetrics and Gynaecology, University of Southern California, Keck School of Medicine, Los Angeles, CA USA; 11 Department of Family Medicine and Public Health, University of California San Diego School of Medicine, San Diego, CA USA

**Keywords:** Breast cancer, Menstrual cycles, Progesterone, Estrogens, Tumor doubling time, DNA replication, WNT4

## Abstract

Many factors, including reproductive hormones, have been linked to a woman’s risk of developing breast cancer (BC). We reviewed the literature regarding the relationship between ovulatory menstrual cycles (MCs) and BC risk. Physiological variations in the frequency of MCs and interference with MCs through genetic variations, pathological conditions and or pharmaceutical interventions revealed a strong link between BC risk and the lifetime number of MCs. A substantial reduction in BC risk is observed in situations without MCs. In genetic or transgender situations with normal female breasts and estrogens, but no progesterone (P4), the incidence of BC is very low, suggesting an essential role of P4. During the MC, P4 has a strong proliferative effect on normal breast epithelium, whereas estradiol (E2) has only a minimal effect. The origin of BC has been strongly linked to proliferation associated DNA replication errors, and the repeated stimulation of the breast epithelium by P4 with each MC is likely to impact the epithelial mutational burden. Long-lived cells, such as stem cells, present in the breast epithelium, can carry mutations forward for an extended period of time, and studies show that breast tumors tend to take decades to develop before detection. We therefore postulate that P4 is an important factor in a woman’s lifetime risk of developing BC, and that breast tumors arising during hormonal contraception or after menopause, with or without menopausal hormone therapy, are the consequence of the outgrowth of pre-existing neoplastic lesions, eventually stimulated by estrogens and some progestins.

## Introduction

The absolute lifetime risk of developing breast cancer (BC) in women in the USA is high and has increased from 1 in 11 (9.1%) in 1975 to 1 in 8 (12·9%) in 2021 [1]. Similar, or even higher, lifetime risks have been reported in Europe, as illustrated for the UK with a risk of 1 in 7 (15%) for women born after 1960 [2]. For men, the lifetime risk of acquiring BC is 1 in 833 (0.1%) in the USA, 100 times lower than for women [3]. A recent article describes that the majority of BC in women (58% to 83%) is related to reproductive factors; another 5–10% of BCs are thought to be caused by hereditary germ-line mutations, with the remaining BCs related to environmental factors [4–8]. Of vital importance in this review is the distinguishment between cause and stimulation, when judging the effect of reproductive, genetic and environmental factors in inducing BC.

An important reproductive determinant of developing BC is the cumulative life-time number of menstrual cycles (MCs) and the accompanying repeated intermittent exposure to progesterone (P4) [9, 10]. Women in developed countries have their first menstrual period (menarche) on average at 11 years of age, and their last menses (menopause) occurs on average at 51 years, 40 years later. With an average MC duration of 28 days, a woman has 13 cycles per year, equating to a total of 520 MCs in her lifetime. Assuming that each year of regular menstrual cycling contributes equally to the BC risk, and that hormone fluxes in MCs are a primary determinant of BC risk, a simple calculation finds that MCs carry a relative risk of BC of about 2.5% per year of regular cycling. A highly significant linear relationship between the BC risk and the cumulative number of MCs before a first full term pregnancy as well as for the life time number of MCs has been reported earlier already [11, 12].

### Search strategy

We reviewed the literature to investigate the relationship in women between the risk of developing BC and reproductive variables, especially the total lifetime number of MCs a women experiences during her life, and the role of P4 as mutagenic factor causing BC under the following circumstances:

1. physiological variations in the number of MCs related to age at menarche, age at menopause, pregnancies and total lifetime duration of lactation;
2. genetic interference with the occurrence of MCs in hypogonadotropic syndromes including the Turner syndrome (TS), primary congenital hypogonadotropic hypogonadism (CHH), the Kallmann syndrome (KaS) and pure gonadal dysgenesis (PGD), also known in males as the Swyer syndrome (SwS);
3. genetic interference with the hormonal levels or MCs in normogonadotropic syndromes including complete androgen insensitivity syndrome (CAIS) and Mayer–Rokitansky–Küster–Hauser (MRKH) syndrome, also known as Müllerian agenesis;
4. pathological conditions affecting the number of MCs with long-term primary or secondary hypo- or hypergonadotropic amenorrhea and absence of MCs due to anorexia nervosa (AN) and weight loss-related amenorrhea (WLRA), primary ovarian insufficiency (POI) or failure (POF), and early premenopausal oophorectomy for ovarian cancer or extensive endometriosis, and
5. pharmaceutical interventions suppressing the MC and steroid hormone treatments affecting the risk of BC.

In addition, we summarize the molecular actions of P4 in healthy breast epithelium that may stimulate BC development and confirm that the role of estradiol (E2) and other estrogens in the origin of BC is negligible, but that estrogens are more likely to stimulate the growth of existing BCs.

## Physiological variations

### Menstrual cycles

**Fig. 1:**
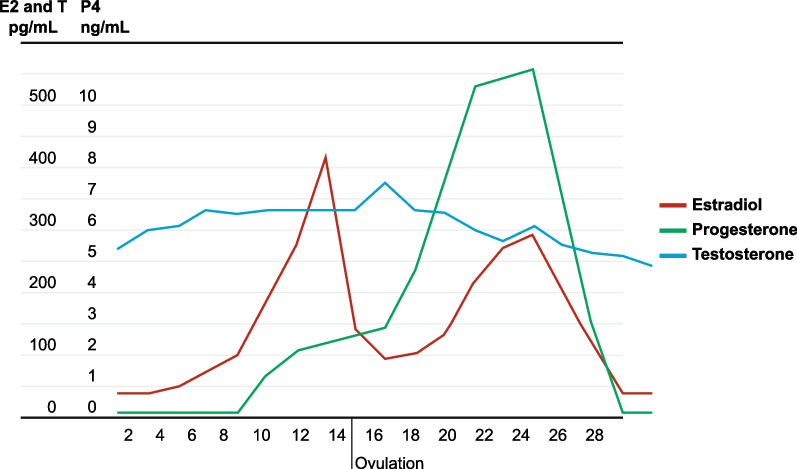
Mean plasma levels of estradiol (E2 pg/mL), progesterone (P4 ng/mL), and testosterone (T pg/mL) during a normal menstrual cycle of 28 days [14, 22]

The average length of a normal MC is 28 days, with a range of 26 to 30 days. A normal MC is characterized endocrinologically by preovulatory and midluteal E2 increases and by P4 secretion during the luteal phase, with P4 levels peaking at the midluteal timepoint. Testosterone (T) levels are stable with a small increase after the preovulatory E2 peak (Fig. 1). When ovulation does not occur in anovulatory cycles, the corpus luteum is not formed and no P4 is synthesized. The primary function of P4, together with E2, is to prepare the endometrium for embryo implantation and support a pregnancy thereafter until term. In addition, during the MC, P4 controls the proliferative effect of E2 on the endometrium, with endometrial shedding and menstrual bleeding occurring when P4 decreases if fertilization does not occur.

Estradiol is essential for ovulation and for the establishment of pregnancy by preparing the endometrium for embryo implantation, while P4 is essential for the establishment and continuation of pregnancy. Testosterone is a precursor of E2, with T levels up to eight times higher than E2 levels (Fig. 1), with large interindividual variations [13, 14]. The function of T in women seems to be primarily related to sexual function, especially desire and arousal, along with a favourable effect on mood and musculoskeletal health [15, 16]. Intermittent exposure to hormones may be more relevant than continuous exposure, since every new hormone peak may initiate a new mutation in breast progenitor and stem cells, and therefore, in this paper we have focused on P4 and E2 and not on T.

### Menarche and menopause

Early menarche and late menopause, which correspond with more MCs, are associated with a small increase in the risk of BC [12]. In the Collaborative Group on Hormonal Factors in Breast Cancer (Collaborative Group) cohort of 118,964 women with an average age at menarche of 13.1 years, there was a 5% increase in lifetime risk of breast cancer for every year younger at menarche [12], so these early cycles carry double the average yearly risk of 2.5%. This may be related to a higher propensity for mutations to arise in the developing breast. A later age of menopause is associated with a 2.8–3.5% increased risk of BC for each additional year of MCs, as demonstrated in another Collaborative Group analysis of data from 51 epidemiological studies [12, 17]. The effects of age at menarche and at menopause on the risk of BC are generally recognized as significant although the increases in absolute risk are small.

### Pregnancy and lactation

**Fig. 2:**
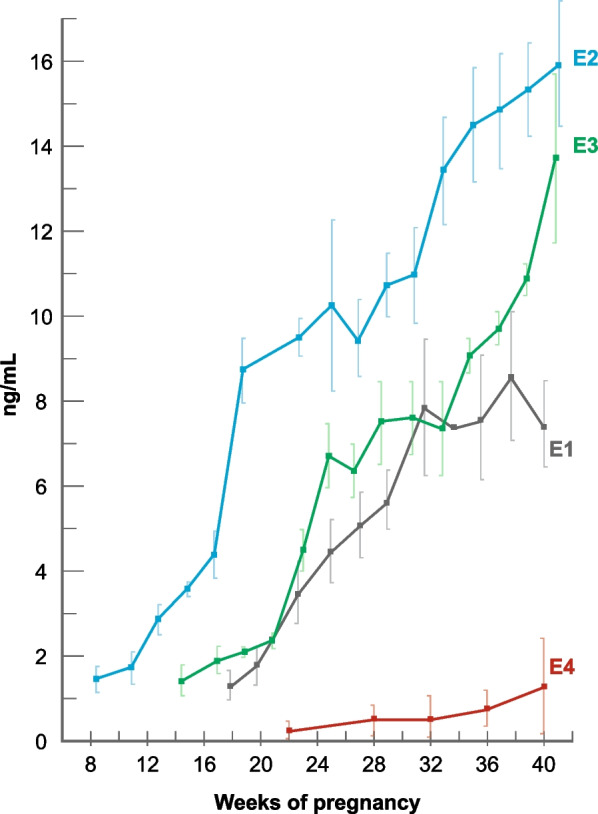
Estrogen levels (ng/mL) during human pregnancy. E1: estrone; E2: estradiol; E3 estriol; E4: estetrol. Adaptation of a figure published by Levitz [119], based on Tulchinsky [120, 121]

The relationship between BC and pregnancy is complicated. Early pregnancy protects against BC, whereas a first pregnancy after the age of 35 increases the risk [18]. Clinically it is well known that pre-existing estrogen receptor positive (ER^+^) tumors may grow rapidly during pregnancy. Data from the Collaborative Group Report in 2002 show a 7% decrease in BC risk with each birth [19]. More recent papers confirm the protective effect of a full term pregnancy [20] that is not found with pregnancies ending in first trimester abortion [21]. A full-term pregnancy, including the post-partum MC recovery, will prevent about one year of MCs, and the associated 7% reduction in BC risk is higher than the hypothetical 2.5% per year without MCs. Progesterone is an absolute requirement for the continuation of pregnancy and P4 levels increase from 5 to 20 ng/mL during the luteal phase of the MC (Fig. 1) to levels of 50–300 ng/mL at term pregnancy [22]. The levels of the four natural estrogens estrone (E1), E2, estriol (E3) and estetrol (E4) also increase progressively during pregnancy (Fig. 2), resulting in very high levels at term pregnancy. The seeming contradiction posed by the association of increased P4 and estrogen levels during pregnancy and reduced BC may be explained by the fact that P4 and estrogen levels increase gradually, contrary to the rapidly changing levels during the MC, associated with mutations.

**Fig. 3:**
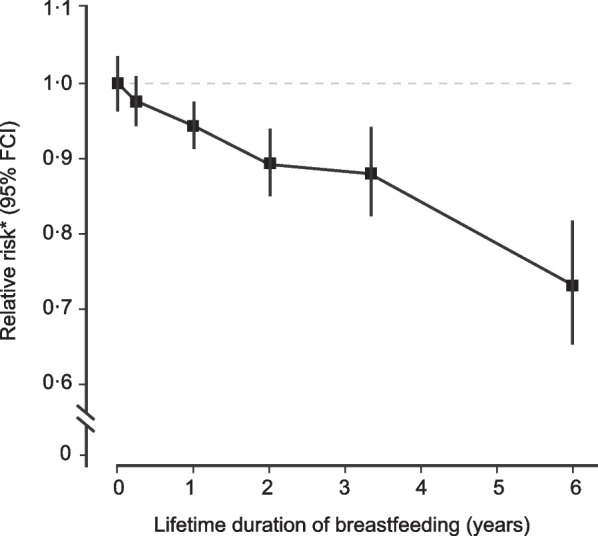
Relative risk of breast cancer in parous women in relation to lifetime duration of breastfeeding [19]. RightsLink Elsevier permission

The absence of MCs during lactation is related to a decreased risk of BC correlating with the lifetime total duration of breastfeeding. Figure 3 shows this effect with a 30% decrease in the relative risk (RR) of BC for a total duration of lactation of 6 years, reflecting a 5% decrease in BC risk per year of lactation [19]. This is double the hypothetical decrease based on the 2.5% BC risk reduction per year without MCs. Also age-related lobular involution of the breast is related to a lower risk of BC [23], whereas benign breast disease is a risk factor for BC [24].

### Demographic data

When multiple pregnancies are combined with long periods of lactation as occurs in societies without contraception, the risk of BC decreases considerably. For example, in the Amish population in the USA this way of life is associated with a standardized BC incidence ratio (SIR) of 0·58 (95% confidence interval [CI] 0·39–0·83) [25], reflecting a reduction in the lifetime BC risk of about 40%.

**Fig. 4:**
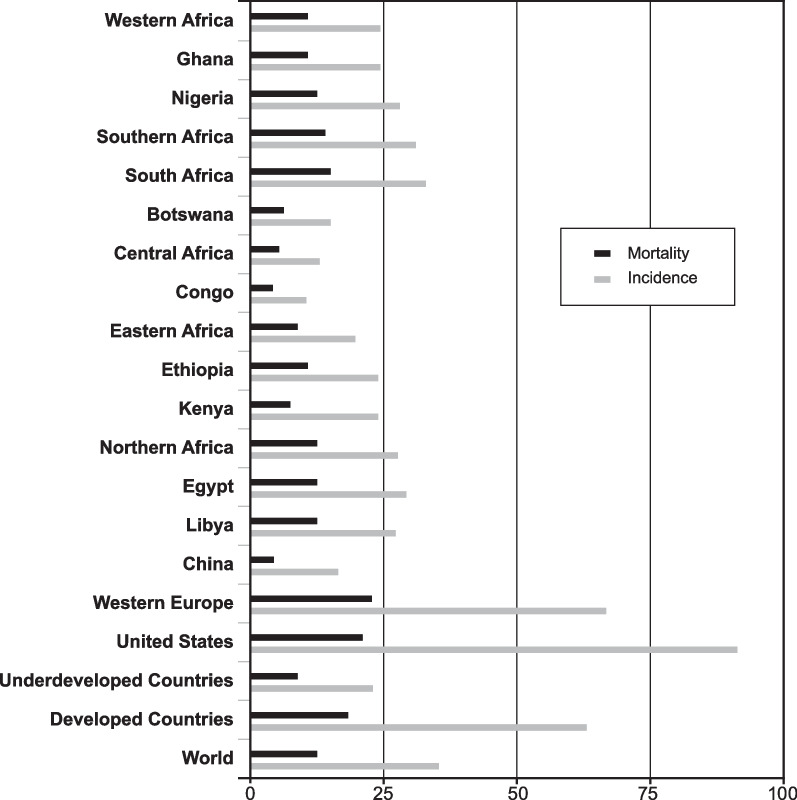
Breast cancer incidence and mortality among women of African nations compared with the USA and other international populations [26]. Rates shown are per 100,000 persons. Open Access reference

The absolute lifetime risk of developing BC for women in Europe, the USA and Asia experiencing over 500 MCs during their life is 13–15%. Women from sub-Saharan Africa have a 4 to fivefold lower incidence of BC compared to economically advantaged western countries with rates of 15–25 per 100,000, versus 70 and 90 per 100,000 for Western Europe and the USA, respectively (Fig. 4) [26]. This is explained by what Tomasetti and co-workers call “the replicative factors related to BC,” including a protective reproductive history, with late menarche, early first pregnancy, high parity with prolonged breastfeeding, irregular infrequent cycles, fewer ovulatory cycles, more stress-related amenorrhea and early menopause [6]. Recent reviews have found that these replicative causes of BC are responsible for 58–83% of BC, with the remainder attributable to genetic and environmental causes [4–8].

## Genetic interference

### Hypogonadotropic syndromes without menstrual cycles

There are a number of genetic conditions interfering with the occurrence of MCs, as reported in detail previously [27]. These genetic abnormalities are hypogonadotropic syndromes including the Turner syndrome (TS), primary congenital hypogonadotropic hypogonadism (CHH), the Kallmann syndrome (KaS) and the pure gonadal dysgenesis (PGD) or Swyer syndrome (SwS). Normogonadotropic genetic syndromes interfering with MCs are the complete androgen insensitivity syndrome (CAIS), and the Mayer–Rokitansky–Küster–Hauser (MRKH) syndrome, also called Müllerian agenesis. The risk of BC is very low or absent in women with TS, KaS, CHH and PGD, who also have reduced breast development, which may contribute to the very low BC risk. Although treatment with estrogens in these genetic disorders stimulates breast development, it seldomly causes BC and is observed only rarely in TS. The CAIS and MRKH syndrome are described in more detail below since both conditions provide insight in the role of P4 in BC.

### Normogonadotropic syndromes without menstrual cycles

The complete androgen insensitivity syndrome (CAIS), previously known as testicular feminization, is an X-linked recessive condition in male genotypes, mostly caused by a defect in the androgen receptor gene, resulting in inactivity of the androgen receptor or by congenital interference with T synthesis. The prevalence of CAIS has been estimated as 1 in 90,000 to 100,000 genomic XY new-borns [28]. In CAIS without functional T, the genotypic male XY embryo develops into a phenotypic female. The external male sexual organs do not develop in spite of hormonally active testicles, located in the abdomen or inguinal canal. There is a short vagina, but regularly no uterus, no oviducts and no ovaries. The testicles synthesize male amounts of T with accompanying T levels, and since T is partially metabolized into active E2, this results in adequate female size and structure breast development [29]. Patients with the CAIS syndrome present with the unique combination of female E2 levels and female size and structure breasts without P4. In individuals with the CAIS syndrome, only a single case of a juvenile fibroadenoma of the breast [30], and no BC has been reported [31].

The Mayer–Rokitansky–Küster–Hauser (MRKH) syndrome or Müllerian agenesis is a congenital malformation characterized by failure of the Müllerian ducts to develop in an XX phenotype female, resulting in the absence of the upper two-thirds of the vagina, the uterus and eventually also the oviducts, all derived from the paramesonephric Müllerian ducts [32]. MRKH occurs in about 1:4000 to 5000 female births [33]. In MRKH women, the karyotype is normal (46, XX), and so is ovarian function and breast development[34]. An important cause of MRKH is the absence of activity of the growth factor WNT family member 4 (WNT4) during a critical period of formation of the internal genitalia, 6 to 8 weeks after conception [34]. As a consequence, the Müllerian ducts fail to develop in the absence of WNT4 [35, 36]. In addition, WNT4 has an essential function in mammary gland development downstream of P4 signalling [37]. In normal male embryos, WNT4 activity is counteracted by the sex-determining region Y (*SRY*) gene on the Y chromosome causing regression of the Müllerian ducts and development of the mesonephric Wolffian ducts, resulting in the formation of epididymis, vas deferens and seminal vesicles in the male [38]. In women with MRKH, only a single case of BC has been reported in the literature [39]. Remarkably, (1) BC was not mentioned at all in a recent MRKH review [40], (2) MRKH patient organizations in the USA (55,000 members), China (130,000 members) and the Netherlands were not aware of any cases of BC among their members when contacted [27] and (3) BC is not mentioned in an extensive document of the American College of Obstetricians and Gynecologists (ACOG) on Müllerian agenesis, which summarizes the relationship between MRKH and many diseases, but does not mention BC [41].

## Pathological effects

Diseases and conditions accompanied by long-term hypo- and hypergonadotropic secondary amenorrhea and absence of MCs include anorexia nervosa (AN) and weight loss-related amenorrhea (WLRA), primary ovarian insufficiency (POI) or failure (POF), and premenopausal oophorectomy in women with an increased genetic risk of BC or ovarian cancer or with extensive endometriosis. Here, we review the evidence regarding the risk of BC in women with these pathological conditions.

### Anorexia nervosa and weight loss-related amenorrhea

Long-term functional hypothalamic amenorrhea may be caused by AN and WLRA. Several large studies have reported a decreased risk of BC in AN. Compared to the Swedish general population, 7303 women hospitalized for AN before the age of 40 years had a 53% (95% CI 3‒81%) lower incidence of BC, and in the parous women the risk was 76% lower (95% CI 13‒97%) [42]. In a retrospective cohort study on the incidence of BC among 22,654 women with AN from Sweden, Denmark and Finland, the incidence rate ratio for BC compared to randomly selected persons from population registers, and adjusted for age, parity and age at first child, was 0.61 (95% CI 0.49‒0.77) [43]. In the Sister Study in the USA, including 47,813 women who had a sister with BC, 3% (1569 women) reported a history of an eating disorder [WLRA, AN or bulimia nervosa (BN)]. Compared to sisters without an eating disorder, the sisters with AN or BN had a reduced BC risk, with a hazard ratio (HR) of 0.62 (95% CI 0.42‒0.92) [44]. Although these studies do not provide details about MCs, primary or long-term secondary amenorrhea is an obligatory symptom for the diagnosis of AN according to the DSM-5 [45]. Therefore, one may assume that the women with AN in those studies have substantially reduced numbers of MCs, and this may be a causal factor associated with the observed significant decrease in BC risk in the order of 50‒75% [42, 43]. The plausibility of this hypothesis is supported by the observation that for all cancers except BC, women with AN have a higher incidence ratio or SIR and a higher standardized mortality rate [43, 46].

### Polycystic ovary syndrome

The BC risk in women with PCOS is not increased, although these women generally have relatively high E2 and T levels. According to the Rotterdam criteria, anovulation is not required for the diagnosis of PCOS, since hyperandrogenaemia and polycystic ovaries are sufficient for the diagnosis [47]. There is a huge amount of data available on BC and PCOS, but quite remarkably, we have not found specific data in the literature on the BC risk in the subgroup of PCOS women with anovulation, who have reduced exposure to P4 [48].

### Primary ovarian insufficiency

Primary ovarian insufficiency (POI) or failure (POF) is generally defined as secondary amenorrhea of at least 12 months duration with elevated follicle-stimulating hormone levels in women younger than 40 years [49–51]. Women with POI experience fewer MCs, and therefore, a lower incidence of BC is expected. Menopause before the age of 49 was associated with a decreased BC risk [52], and this effect was observed in Caucasian as well as in Asian women. In a study conducted among 12,134 Dutch women, whose menopause occurred before the age of 40 years, a significantly increased total all-cause mortality was observed after adjustment for confounding factors, as compared with the reference group whose menopause occurred at age 50–54 years [HR 1.40 (95% CI 1.15–1.17)] [53]. However, of note, the corresponding incidence of mortality due to BC was much lower than in the reference group [HR 0.55 (95% CI 0.17–1.82)] [53]. In a Dutch population-based BC-screening cohort of 10,591 women, early menopause (occurring at ages below 45, or at 45–49 years) also showed a protective effect on the risk of BC [HR 0.66 (95% CI 0.43–0.91)] and [HR 0.67 (95% CI 0.48–0.94)], respectively, as compared with menopause occurring at age 55 or older [54]. For example, women who became menopausal below 44 years of age had a 34% lower risk of BC than women aged 55 years. The annual hazard rate in relation to the BC incidence decreased by 2.6% in women who had an earlier menopause [54], which aligns with our calculation of a 2.5% BC risk reduction per year without MCs. In Chinese women, POI was associated with an increased risk of all-cause and cancer mortality [HR 1.29 (95% CI 1.08–1.54) and HR 1.38 (95% CI 1.05–1.81), with or without menopausal hormone therapy (MHT), respectively] [52]. However, POI was inversely associated with the incidence of BC [odds ratio [OR] 0.59 (95% CI 0.38–0.91)]. BC was not addressed at all in a recent review on POI [55], as well as in the recent International Menopause Society white paper on POI [56].

### Premenopausal oophorectomy

Prophylactic premenopausal mastectomy and oophorectomy are used widely to prevent BC in women who carry genetic mutations that predispose to an increased risk of BC, such as mutations in the *BRCA1* and *BRCA2* genes [57]. Risk-reducing salpingo-oophorectomy was associated with a statistically significant reduction in the risk of BC in the combined group of *BRCA1/2* mutation carriers [HR 0.49 (95% CI 0.37–0 65)]. Similar risk reductions were observed in *BRCA1* mutation carriers [HR 0.47 (95% CI (0.35–0.64)] and in *BRCA2* mutation carriers [HR 0.47 (95% CI 0.26–0.84)] [57]. Bilateral oophorectomy for the treatment of ovarian cancer is also associated with a large reduction in BC risk [58]. It would be interesting to know the BC risk after bilateral oophorectomy for extensive endometriosis, but we have not found this information in the literature.

## Pharmaceutical effects

The present review is focusing on the effect of the steroid hormones of the natural menstrual cycle (MC) on the risk of breast cancer (BC). The effect of the BC risk of pharmaceutical treatments with exogenous reproductive steroid hormones for hormonal contraception (HC), endometriosis treatment, hormone treatment of transgender individuals and menopausal hormone therapy (MHT) is beyond the scope of this review and deserves a detailed separate analysis. Here we will only briefly summarize the effect of HC, including P-only, on E-only MHT and on transgender treatments.

### Menstrual cycle suppression and breast cancer risk

Hormonal contraception (HC) and endocrine treatment of endometriosis are major pharmaceutical applications in Women’s Health, where the natural MC is suppressed and where the relationship between the administration of estrogens and progestogens (including P4 and progestins) and the occurrence of BC has been extensively studied. To judge this relationship, one has to realize that it takes about a decade from a stem cell mutation in the breast until the tumor has grown to a size enabling the diagnosis of a BC [59]. This means that tumors diagnosed during the first decade of HC and endometriosis treatment are most likely due to mutations that occurred during earlier spontaneous MCs as are BCs diagnosed during the first decade after menopause with or without MHT. In case ovulatory MCs would be responsible for the high BC incidence in women indeed, MC suppression by combined E/P oral contraceptives, ovulation inhibiting progestin-only (P-only) contraception or hormonal endometriosis treatment, would protect against BC and one would expect to observe a lower incidence of BC. However, the data show the contrary [60–63]. As an example, Table 1 shows the results in a recent large Danish HC study including some P-only contraceptives, confirming no decrease in BC and even an increase with duration of use [64].

**Table 1:** Relative risk (95% CI) of breast cancer, observed in a Danish cohort of women aged 15–49 years and without a history of cancer, venous thromboembolism or fertility treatment [64]

(a) Relative risk according to time since use and duration of any type of hormonal contraception			
Duration of use	Relative risk of breast cancer (95% CI)		
	< 1 yr since recent use	1 to < 5 yr since recent use	5–10 yr since recent use
< 1 yr	0.96 (0.78–1.19)	0.96 (0.85–1.09)	1.01 (0.88–1.15)
1 to < 5 yr	1.04 (0.88–1.23)	1.06 (0.96–1.18)	1.07 (0.94–1.20)
5–10 yr	1.33 (1.11–1.59)	1.16 (1.02–1.33)	1.30 (1.06–1.58)
> 10 yr	1.52 (1.17–1.98)	1.16 (0.89–1.49)	NA

(b) Relative risk among women using current or recent P-only contraception		
Current or recent P-only contraception		Relative risk (95% CI)
Oral contraception	NETA	1.00 (0.80–1.25)
	LNG	1.93 (1.18–3.16)
	DSG	1.18 (0.87–1.60)
Intra-uterine device	LNG	1.21 (1.11–1.33)

Reference group: women who had never used hormonal contraception; Recent: defined as discontinuation of hormonal contraception within the previous 6 months; NA: not applicable due to too small number of events

*CI* confidence interval, *DSG* desogestrel, *LNG* levonorgestrel, *NETA* norethisterone acetate, *yr* year

### Estrogen-only MHT in the WHI study and breast cancer risk

A special case is the Women’s Health Initiative (WHI) study, the largest randomized controlled MHT trial ever, which nearly abolished MHT use forever. Table 2 summarizes the BC results of the WHI study. This study is different from many others, since conjugated equine estrogens (CEE) plus medroxyprogesterone acetate (MPA) (E/P) and E-only treatment started on average 12 years after menopause in women with a mean age of 63 years (range 50–79) at the time of enrollment. This late start of MHT is highly relevant, since it means that in most cases already existing BCs had been devoid of estrogens for more than 5 years, so those BCs may have become sensitive to the therapeutic effect of (high dose) estrogen treatment which in this setting, and in the absence of potentially BC stimulating progestins, causes tumor regression (4–8). Initially, the E/P arm of the study was thought to have an increased BC risk which was of concern despite the lack of statistical significance [65]. Retrospectively, this small non-significant increase was likely due to unmasking of existing BCs by the proliferative influence of MPA. Furthermore, later analysis demonstrated that the risk difference in the E/P arm was based on a lower BC risk in women with any prior hormone use [66]. In contrast, from the very beginning, the E-only arm of the WHI showed protection against BC [67], exactly what would be expected, based on the long previous period of estrogen deficiency [4–8]. The most important WHI follow-up paper was the one on long-term all-cause and cause-specific mortality published in JAMA in 2017, summarizing a cumulative follow-up of 18 years [68]. It was found that the median treatment of 5.6 years with the E/P combination and of 7.2 years with E-only in this older age group of postmenopausal women was not associated with an increased risk of all-cause, CV or cancer mortality 18 years later. In the pooled analysis combining E/P and E-only for women 50 to 59 years old when they entered the study, there was a 31% reduction in all-cause mortality [HR 0.69 (95% CI 0.51‒0.94)]. The HR for death due to BC for the E/P combination used in the WHI study was elevated but not statistically significant [1.44 (95% CI 0.97‒2.15)], suggesting a stimulatory, and possibly mutagenic effect of MPA. However, the most impressive BC outcome in this analysis was the highly significant finding that death due to BC (and not just risk) in the E-only CEE group was 45% lower than in the placebo group with a HR of 0.55 (95% CI 0.33‒0.92) (Table 2) [68]. The conclusion is that E-only MHT extends life when MHT is started at least 5 years after menopause (in the WHI study on average at 63 years).

**Table 2:** Risk of breast cancer (95% CI) over time in women receiving estrogen-only (CEE) or estrogen (CEE) plus progestin (MPA) menopausal hormone treatment (MHT) in the Women’s Health Initiative (WHI) study

Study	Overall BC risk	Cumulative BC risk	Statistical analysis
WHI CEE (Anderson 2004) [117] Mean FU 6.8 yr	Overall HR 0.77 (0.57–1.06)	Cumulative Hazard (8 yr) HR 0.77 (0.59–1.01)	HR (adjusted) vs Placebo
WHI CEE (Chlebowski 2020) [118] Median FU 16.2 yr		Cumulative Hazard (22 yr) HR 0.78 (0.65–0.93)	*P* = 0.005 versus placebo
WHI CEE/MPA (Rossouw 2002) [65] Mean FU 5.2 yr	Overall HR 1.26 (0.83–1.92)	Subgroup FU ≥ 10 yr HR 1.81 (0.60–5.43)	HR (adjusted) versus Placebo *Z* score for trend 2.56
WHI CEE/MPA (Hodis 2018) [66] Mean FU 5.6 yr	HT naïve patients (75% of cohort) HR 1.02 (0.77–1.36)*	Subgroup with prior HT HR 1.96 (1.17–3.27)	HR versus Placebo Difference naïve vs prior HT: *P* = 0.027
WHI CEE/MPA (Chlebowski 2020) [118] Median FU 18.9 yr		Cumulative Hazard (22 yr) HR 1.28 (1.13–1.45)	*P* < 0.001 versus placebo
Study	BC mortality		
WHI CEE (Manson 2017) [68] Cumulative FU 18 yr	Overall HR 0.55 (0.33–0.92)		*P* = 0.02 versus placebo
WHI CEE/MPA (Manson 2017) [68] Cumulative FU 18 yr	Overall HR 1.44 (0.97–2.15)		HR (adjusted) versus Placebo

*CEE* conjugated equine estrogens, *CI* confidence interval, *FU* follow-up, *HR* hazard ratio, *MPA* medroxyprogesterone acetate, *WHI* Women’s Health Initiative, *yr* years

*Comment authors: an increased risk of de novo development of breast cancer during the mean 5.6 years of randomized treatment in the WHI CEE/MPA trial is biologically implausible (Santen [59])

### Hormonal treatment of transgender individuals

Both male-to-female (MtF) and female-to-male (FtM) transgender individuals are treated long-term with high doses of, respectively, estrogens and androgens, and the consequences of these steroid hormone treatments have been reported by us in detail previously [27]. Here we summarize the data relevant for this review.

#### Estrogen treatment of MtF transgenders and BC risk

The MtF gender identity disorder occurs in about 1 in 12,000 born males [69]. These individuals are treated with estrogens for breast development and with antiandrogens or orchidectomy to suppress androgens. Adequate breast development with normal histology is obtained by treatment with high doses of estrogens (HDE) and is continued lifelong [70, 71]. In a study in 2307 MtF transgender individuals, two cases of BC have been reported after exposure to HDE for 5 to 30 years, resulting in an incidence of 4.1/100,000 person-years [70]. A study in MtF transgender individuals in the USA found similar results [72]. A recent nationwide cohort study in the Netherlands reported 15 cases of BC in 2,206 MtF transgenders on HDE treatment with a mean duration of 18 years (range, 7–37 years). Compared to Dutch males, the overall risk of BC was increased [SIR 46.7 (95% CI 27.2–75.4)], but the risk was still much lower than in the female population [SIR 0.3 (95% CI 0.2–0.4)] [73]. These studies show that the incidence of BC in MtF transgenders with fully developed normal breasts, treated long term with HDE without progestins (since they have no uterus), is much lower than the BC risk of cisgender females and somewhat higher than the BC risk of cisgender males [70–73].

#### Testosterone treatment of FtM transgenders and BC risk

The FtM gender identity disorder occurs in about 1 in 30,000 born females [69] and concerns genetic and phenotypic females, who feel male and wish to become phenotypic males. In these patients the ovaries, the uterus and the breasts are surgically removed and masculinization is achieved by treatment with high doses of testosterone (HDT). The occurrence of BC in FtM transgenders depends on the completeness of removing all mammary tissue by mastectomy [70, 72, 74] and is comparable to the prevention of BC by mastectomy in high-risk women carrying germline mutations such as *BRCA1* and *BRCA2* [75]. In the Dutch cohort study, four cases of BC were reported in 1,229 FtM individuals treated with HDT with a SIR vs Dutch cisgender women of 0.2 (95% CI 0.1‒0.5) [73]. In a systematic review of BC in HDT-treated FtM transgender individuals, 17 cases of BC were found originating from eight studies [76], resulting in a BC risk somewhat higher than in cisgender males. These studies show that long-term HDT treatment does not increase the risk of BC significantly.

## Carcinogenicity of progesterone

The mammary gland is a highly heterogenous tissue with many different cell lineages of which the differentiation hierarchy has not yet been definitively defined [77]. The adult breast epithelium of the mammary gland contains differentiated ductal, alveolar and myoepithelial cells, and their progenitors. In addition, cells that provide long-term maintenance and regenerative potential, such as multipotent mammary stem cells (MaSCs) and certain short- and long-term progenitors, have been identified [78–82].

While most of these cells, including the stem cells, do not express the ER and progesterone receptor (PR) [77], it has now been well established that E2 and P4 exert their effects on the breast epithelium by inducing the secretion of paracrine factors by a relatively small pool of ER^+^/PR^+^ cells [80].

**Fig. 5:**
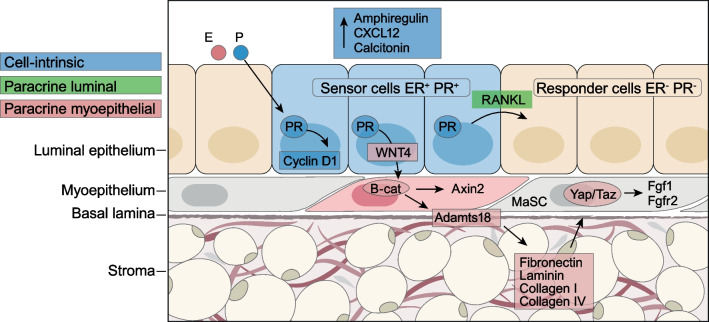
The progesterone signaling hub in the adult mammary epithelium progesterone, upon binding to its receptor in the ER^+^/PR^+^ sensor cells (blue) activates different signaling pathways. It can stimulate cell-intrinsic proliferation by a cyclin D1-dependent mechanism (blue) and induce secreted factors like Amphiregulin, CXCL12, or Calcitonin (blue). Distinct PR^+^ cells induce WNT4, which acts on the myoepithelium where it activated canonical WNT signaling, which results in the expression of the secreted protease Adamts18 that cleaves fibronectin. As a result the ECM, part of the stem cells niche is biochemically altered with resulting activation of the hippo signaling pathway and increased transcription of FGFR signaling components (red). In other PR^+^ cells, RANKL is induced that induces the proliferation of neighboring ER^−^/PR^−^ responder cells (green) [84]. Open Access reference and author permission

During puberty, the mammary gland undergoes phenotypic and morphological changes, particularly regulated by E2 and to a lesser extent by P4 [83]. However, when female sexual maturity is reached around age 15–17 years, the profound phenotypic changes of the breast epithelium during each MC are mainly induced by P4 [84]. In the follicular phase of the MC, when E2 peaks, ER^+^ cells upregulate the expression of the PR to increase the pool of PR^+^ cells, which hardly induces any morphological or molecular changes in the epithelium [85, 86]. When the MC enters the luteal phase, the increasing P4 levels initially induce PR-mediated proliferation of PR^+^ cells (intrinsic effect), and subsequently of neighboring hormone receptor negative (HR^−^) cells (extrinsic), including epithelial progenitor cells and MaSCs (Fig. 5) [84, 85]. The cell intrinsic effect is mediated by cyclin D1 and has an overall mild proliferative effect on the breast epithelium, while the extrinsic stimulation of adjacent HR^−^ cells, including MaSCs, by the activated PR^+^ cells has a much more profound effect. The latter is largely mediated by the paracrine factors RANKL and WNT4, which are direct targets of the PR and play important physiological roles in the breast [87, 88]. Both factors have been shown to be strongly upregulated during the luteal phase in the breast epithelium of healthy premenopausal women, concomitant with genes involved in the cell cycle and DNA replication [86]. The P4-mediated induction of RANKL and WNT4 and the consequent activation and proliferation of MaSCs has been linked to BC initiation [89–91]. As stem cells are considered an important cancer cell-of-origin in BC [92], and proliferation (or DNA replication) is a prominent source of mutations in cancer [6], P4 is likely to impact breast carcinogenesis [93].

Breast cancer has been shown to be a type of cancer largely driven by DNA replication errors, with up to 83% of cases possibly attributable to this factor [6]. Such errors are caused by molecular processes that generate particular mutational signatures/patterns in DNA [94], which have also been identified in the DNA of breast tumors [95–98]. This revealed that in BC, one of the most important sources of somatic mutations arising during DNA replication stems from the activity of APOBEC (Apolipoprotein B mRNA editing enzyme catalytic) enzymes, which deaminate, and thereby convert, cytosine nucleotides [99].

One of the members of this family of enzymes, APOBEC3B, has been shown to be expressed and to actively generate mutations in breast tumors [100, 101]. APOBEC3B expression strongly correlates with the cell cycle and with the DNA damage response pathway in BC [102]. It introduces mutations during DNA replication, and therefore confers or increases replication stress [99]. Interestingly, this enzyme was shown to be statistically significantly upregulated during the luteal phase in breast epithelium of healthy premenopausal women [86]. This suggests that under the influence of P4, during each MC, increased activity of this enzyme can introduce DNA mutations in breast epithelial cells to increase BC risk. Activity of APOBEC3B has been linked to the generation of mutations in TP53 [100], an important early driver in BC [103]. In addition, APOBEC-mediated mutational signatures have been observed in normal bronchial, colon, oesophageal and bladder epithelium [104–107].

The proliferative potential of the breast epithelium during the MC decreases with age [108, 109], and after menopause, when exposure to intermittent levels of P4 stops and the levels of E2 decline, it enters a quiescent state. If P4 is an essential component in breast carcinogenesis, how does this hormone impact the increased BC incidence observed in postmenopausal women aged > 60 years? It is plausible that the period between the first MC and menopause is an important source of somatic mutations accumulating in breast epithelial stem cells, a process P4 appears intricately to be involved in.

Studies into the genomic evolution of BC reveal that tumors evolve through several developmental stages before they are diagnosed [96]. Two pre-diagnosis stages are described, of which the first one is by far the longest and is suggested to take decades and slowly accumulates many DNA alterations [103]. The second stage unfolds over a relatively short timeframe (months to a few years) and is postulated to contribute the larger part to tumor size [95]. Therefore, epithelial stem cells that have acquired mutations during the reproductive years as a consequence of P4 action may remain dormant for an extended period of time after menopause. Under the influence of a gradually changing breast tissue microenvironment [110] and perhaps affected by changes in immune competency, pre-malignant lesions may only become evident at an advanced age after menopause.

## Considerations

### Lifetime number of menstrual cycles and breast cancer risk

We have reviewed the available information on the effect of physiological, genetic, pathological and pharmaceutical variations on the number of MCs, the presence of P4 and E2 peaks, and the risk of BC. We confirm the high correlation reported earlier between the number of MCs and the risk of BC [11, 12]. Early menarche and late menopause are related to a small increase in BC risk, proportional to the higher number of MCs with an even somewhat higher risk of early menarche, which may be due to the higher sensitivity of the developing breasts. Pregnancy and lactation reduce the risk of BC, and the yearly risk reduction of, respectively, 7% and 4.3% is somewhat higher than the expected 2.5% per year based on the absence of MCs alone, suggesting additional protective mechanisms. The substantially lower number of MCs in women with pathological reductions in MCs by AN/WLRA, POI and after premenopausal oophorectomy is accompanied by a large and proportional reduction in the lifetime BC risk of approximately 50% [42, 43, 54, 57], confirming the MC hypothesis.

### Genetic interference with menstrual cycles and breast cancer

The risk of BC appears to be very low or absent in individuals with genetic abnormalities interfering with the occurrence of MCs such as TS, KaS, CHH and PGD. Generally, these individuals have little breast development and breast size may have some effect on the BC risk [111], although in normal women no appreciable association between breast size and BC has been found [112]. E/P treatment in individuals with these genetic disorders induces normal breasts, but rarely causes BC. Mastectomy performed in women with BC-related mutations such as *BRCA1* and *BRCA2*, completely prevents the occurrence of BC [75].

Most remarkable and important are the observations in individuals with the CAIS syndrome and the MRKH syndrome. The genotypic male but phenotypic female individuals with the CAIS syndrome have no BC in the presence of substantial estrogen levels and normal female breasts, which could be explained by the absence of P4. An exception to the relationship between MCs, P4 and BC is the apparent absence of BC in women with the MRKH syndrome, who have ovulatory cycles, sometimes as part of PCOS like endocrinology [113], with accompanying normal P4 levels and normal breasts, but no BC. We speculate that this is related to the absence of the WNT4 gene, also responsible for the MRKH syndrome itself, negating P4 induced WNT4 activity. Further research focusing on the role of WNT4 as a causal factor of BC seems relevant.

### Progesterone as a causal factor of breast cancer

The major reproductive steroid hormones from the normal menstrual cycle E2, P4 and T have all been implicated as causal as well as stimulatory agents of existing BC. As data have shown that P4 has a much more pronounced effect on the healthy breast epithelium during the MC as compared to E2 and T, we have focused on P4 as causal agent and we have carefully tried to distinguish between cause and stimulation. Based on the BC risk in different populations reviewed in this paper, we found that the lifetime number of MCs and the frequent intermittent exposure to P4 during these MCs are major explanations for the high BC risk in women. The repeated intermittent stimulation of proliferation of the breast epithelium by P4 likely contributes to the accumulation of mutations in certain long-lived cells. Breast cancer may develop because of these mutations, and based on a doubling time of the tumor of about 10–12 years it may take a decade until a BC is large enough to be diagnosed [59]. Breast (mammary) tissue has an increased risk of DNA damage compared to other tissues in the human body due to the extensive remodeling in the breast through a high rate of proliferation, apoptosis and differentiation, occurring throughout a woman’s life. Reproductive events responsible for this enormous remodeling are related to a woman’s reproductive history and include puberty, MCs, pregnancy, lactation and postmenopausal involution. We have investigated the relationship between the risk of developing BC and the number of MCs and the accompanying intermittent luteal phase P4 increases that a woman experiences during her lifetime. The repeated intermittent effect of hormone increases may be more relevant than continuous exposure, since every new hormone peak may cause new mutations and contribute to the accumulation of mutations in long-lived mammary progenitor and stem cells.

The relationship between endogenous P4 and BC has been reviewed recently by Trabert et al. [93]. They found no direct association of circulating P4 levels with BC risk, which may not be surprising in the light of our hypothesis, that intermittent P4 peaks are associated with breast carcinogenesis. They also concluded that preclinical and clinical evidence supports differentiation and proliferative roles of P4 in the adult breast primarily through paracrine actions and points to the importance of understanding the role of P4 in controlling the fate of epithelial cells and early events in breast carcinogenesis.

### Environmental factors and breast cancer

The three major causes of BC are generally distinguished as (1) reproductive factors related to a woman’s reproductive history, (2) germline mutations and (3) environmental effects. Contemporary literature suggests that at least 58% and up to 83% of BCs are caused by reproductive factors [4–8]. Our analysis of clinical data strongly suggests that the essential reproductive factor associated with BC is the frequent intermittent exposure to P4 from ovulatory MCs. Crucial is that we have found that BCs hardly ever occur without MCs and without P4, no matter whether environmental factors are present or not. Also, high exposure to E-only in the WHI E-only study and in MtF transgender individuals and high exposure to T-only in FtM transgender individuals is not related to a significant risk of BC, again unrelated to the presence or absence of environmental factors. This suggests that the significance of environmental factors such as exposure to chemicals, alcohol, toxic food contaminants, estrogen-related effects such as high breast density, high E2 levels and exposure to estrogen metabolites, and lifestyle factors such as overweight and (lack of) exercise [5, 7] may have been overemphasized as cause of BC although these factors may certainly stimulate existing BC, which may explain the confusion. The increased incidence of BC in women with high breast density may be related to higher estrogen levels. The increased BC risk related to higher levels of E2, to other non-ovarian estrogens and to estrogen metabolites can all be explained by stronger stimulation of existing BCs. The lower BC risk observed in vigorously exercising female athletes may be related to anovulation and amenorrhea [114, 115], so less P4 with a lower risk of causing BC and less E with less stimulation of existing BC. Also exercise may decrease the body mass index (BMI), and thereby, less estrogens and less estrogen metabolites will be generated from fatty tissue with less BC stimulation as a consequence [116].

Most data on the relationship between environmental factors and the risk of BC are derived from observational studies, and prospective controlled studies on these factors are rare and difficult to perform. Furthermore, except for toxic mutagenic agents such as alcohol, the mutagenicity and mode of action of environmental factors in causing BC are unknown and stimulation of existing BC seems more likely than induction of new BCs. A more detailed analysis of the environmental factors is beyond the scope of this review and deserves a separate analysis.

### Key points

The key points resulting from review are presented in Table 3. The relationship between MCs and BC, and of P4 and BC, is summarized in Table 4, including the P4/WNT4 hypothesis based on the MRKH data.

**Table 3:** Key points

Women experience approximately 500 menstrual cycles (MCs) during the 40 years of their reproductive life, and there is a high correlation between the actual number of MCs and the lifetime risk of developing breast cancer (BC)	
A reduction in MCs due to physiological, genetic and pathological effects reduces the BC risk in proportion with the decrease in the number of MCs	
Progesterone (P4), rather than estradiol (E2), profoundly stimulates proliferation of the breast epithelium during the luteal phase of the MC through the paracrine factors WNT4 and RANKL and by inducing the expression of the ‘DNA mutator’ APOBEC3B, thereby enhancing the risk of developing BC	
Estrogens (E) hardly stimulate normal estrogen receptor positive (ER^+^) breast epithelial cells, but have a strong proliferative effect on neoplastic ER^+^ cells

**Table 4:** Relationship between menstrual cycles, progesterone and breast cancer

*Data supporting the MC hypothesis (includes P4 and E2)*	
Individuals without MCs due to genetic abnormalities appear to have no BC	
Strong correlation between the reduction in MCs due to physiological, genetic and pathological effects (AN, POI) and a decrease in the lifetime risk to develop BC	
Artificial MCs induced by COCs carry a comparable or even somewhat higher BC risk compared to the natural MC	
*Data supporting the specific P4 hypothesis*	
P4 is mutagenic for the breast, while estrogens and testosterone are not	
Individuals with the CAIS syndrome have substantial E2 levels and female size and structure breasts, but no P4 and no BC	
Individuals with the MRKH syndrome have no uterus and no BC, but normal P4, which could be explained by the absence of WNT4 gene activity	
P-only contraception without endogenous ovarian estrogens carries a BC risk comparable to the natural MC or even somewhat higher	
Estrogen-only MHT decreases BC incidence and mortality when started more than 5 years after menopause	
High dose estrogen treatment of MtF transgenders induces normal female size and structure breasts with a very low BC risk	
FtM transgenders treated long term with high doses of testosterone have a low BC risk which is comparable to cisgender males	

*AN* anorexia nervosa, *BC* breast cancer, *CAIS* complete androgen insensitivity syndrome, *E2* estradiolm, *MC* menstrual cycle, *P4* progesterone, *POI* primary ovarian insufficiency, *WNT4* WNT family member 4, *MHT* menopausal hormone therapy, *MtF* male to female, *FtM* female to male

## Concluding remarks

Our analysis of clinical and molecular data suggests that P4 and not E2 or T is an important cause of BC by stimulating proliferation of normal breast epithelium during the luteal phase of the MC through the paracrine factors WNT4 and RANKL. Progesterone also appears to upregulate the expression of the DNA mutator enzyme APOBEC3B during this phase of the MC. These effects of P4 likely increase the accumulation of mutations in long-lived mammary stem and progenitor cells. Estrogens, testosterone (as precursor of E2) and most environmental factors that are related to the risk of BC may stimulate the growth of already existing ER^+^/PR^+^ BC, but have only mild proliferative effects on normal breast epithelium and, in light of our hypothesis, are therefore unlikely to cause BC. We propose to develop medical strategies in women that avoid exposure to natural P4 and progestins as much as possible.

## Data Availability

All data generated or analyzed during this study are included in this published article.

## Abbreviations

ACOG: American College of Obstetricians and Gynaecologists
AN: Anorexia nervosa
APOBEC: Apolipoprotein B mRNA editing enzyme catalytic
BC: Breast cancer
BN: Bulimia nervosa
BRCA1/2: Breast cancer genes 1 and 2
CAIS: Complete androgen insensitivity syndrome
CEE: Conjugated equine estrogens
CHH: Congenital hypogonadotropic hypogonadism
CI: Confidence interval
DSG: Desogestrel
E: Estrogen
E1: Estrone
E2: Estradiol
E3: Estriol
E4: Estetrol
E/P: Estrogen plus progestin
ER: Estrogen receptor
FtM: Female to male
FU: Follow-up
HC: Hormonal contraception
HR: Hazard ratio
HT: Hormone treatment
KaS: Kallmann syndrome
LNG: Levonorgestrel
MaSCs: Mammary stem cells
MC: Menstrual cycle
MHT: Menopausal hormone therapy
MRKH: Mayer–Rokitansky–Küster–Hauser syndrome
MPA: Medroxyprogesterone acetate
MtF: Male to female
NETA: Norethisterone acetate
OR: Odds ratio
P-only: Progestogen-only
P4: Progesterone
PCOS: Polycystic ovary syndrome
PGD: Pure gonadal dysgenesis
POF: Primary ovarian failure
POI: Primary ovarian insufficiency
RR: Relative risk
SIR: Standardized incidence ratio
SRY: Sex-determining region Y gene
SwS: Swyer syndrome
T: Testosterone
TS: Turner syndrome
USA: United States of America
WHI: Women’s Health Initiative
WLRA: Weight loss-related amenorrhea
WNT4: WNT family member 4
yr: Year
